# Thrombospondin-2 in Cardiovascular Disease: Molecular Mechanisms, Biomarker Potential, and Therapeutic Perspectives

**DOI:** 10.3390/cells15131162

**Published:** 2026-06-26

**Authors:** Karolina L. Stępień, Malwina Botor, Jakub Karliński, Alicja Kazik, Grzegorz Machnik

**Affiliations:** 1 Department of Molecular Biology, Faculty of Medical Sciences in Katowice, Medical University of Silesia, Medykow 18 Street, 40-752 Katowice, Poland; mbotor@sum.edu.pl (M.B.); gmachnik@sum.edu.pl (G.M.); 2 Students’ Scientific Society, Department of Molecular Biology, Faculty of Medical Sciences in Katowice, Medical University of Silesia, 40-752 Katowice, Polands88167@365.sum.edu.pl (A.K.)

**Keywords:** thrombospondin-2, extracellular matrix, cardiovascular diseases, biomarker

## Abstract

Thrombospondin-2 (TSP-2) is an extracellular matrix glycoprotein involved in angiogenesis, vascular remodeling, cell adhesion, and tissue repair. Its expression is induced by pathological stimuli, including mechanotransduction, hypoxia, and TGF-β signaling, and has been associated with several cardiovascular diseases (CVDs), such as heart failure, coronary artery disease, abdominal aortic aneurysm, and hypertension. Elevated circulating TSP-2 levels, particularly in combination with NT-proBNP, as well as alterations in THBS2 and its regulatory non-coding RNAs, have been linked to disease severity and adverse cardiovascular outcomes. This review summarizes current evidence on the role of TSP-2 in cardiovascular pathophysiology and its involvement in cardiovascular homeostasis. Although accumulating data suggest that TSP-2 may have diagnostic, prognostic, and therapeutic relevance, its clinical utility as a biomarker or therapeutic target has not yet been established. Further large-scale studies and standardized assessment methods are required to validate its potential and support future clinical translation.

## 1. Introduction

Thrombospondins (TSPs) are a family of matricellular glycoproteins involved in the regulation of cell–cell and cell–matrix interactions, contributing to tissue homeostasis, repair, and remodeling. Structurally, the family consists of five members divided into two subgroups: group A (TSP-1 and TSP-2), composed of trimeric molecules, and group B (TSP-3, TSP-4, and TSP-5), whose members assemble into pentameric complexes [1,2]. These structural differences translate into distinct functions across physiological and pathological conditions (Table 1) [2].

TSPs were first identified in the early 1970s on the surface of activated platelets using SDS-PAGE–based methods [3,4]. Since the characterization of TSP-1 as the first member, additional thrombospondins (TSP-2 to TSP-5) have been discovered, each exhibiting unique patterns of expression in cardiovascular, musculoskeletal, and connective tissues [4,5]. Early studies positioned TSPs as extracellular matrix (ECM) components, but subsequent evidence revealed that they are potent regulators of vascular biology and cardiac remodeling.

Growing interest in TSPs stems from their involvement in key processes central to cardiovascular disease (CVD). TSPs modulate platelet aggregation, fibrin formation and degradation, endothelial adhesion and migration, and cellular proliferation cycles [6]. Additionally, they participate in angiogenic regulation, ECM turnover, and fibrotic signaling. Of particular relevance to cardiology are their interactions with receptors such as CD36, CD47, and integrins, as well as their ability to activate latent transforming growth factor-β (TGF-β), a major driver of myocardial fibrosis and adverse ventricular remodeling [7,8].

Among all members of the family, TSP-2 has emerged as a molecule of exceptional significance in cardiovascular pathophysiology [7]. TSP-2 is highly expressed in the ECM, developing vasculature, and adipose tissue, acting as an anti-angiogenic and pro-fibrotic regulator with essential roles in matrix assembly and tissue repair. Experimental studies demonstrate that genetic deletion of TSP-2 increases susceptibility to cardiac rupture following blood pressure overload, suggesting a protective role in maintaining myocardial structural integrity. Clinically, elevated TSP-2 levels have been consistently associated with adverse outcomes in patients with heart failure (HF), where they reflect the intensity of fibrotic remodeling and correlate with ischemic risk and disease progression in large-scale cohort studies [9]. Despite growing evidence linking thrombospondins, particularly TSP-2, to conditions such as HF, coronary artery disease (CAD), hypertension, and abdominal aortic aneurysm (AAA), significant knowledge gaps still remain uncovered. These include an incomplete understanding of TSP-2–mediated signaling pathways in cardiac remodeling, limited data on its utility as a biomarker across diverse CVD phenotypes, and unanswered questions regarding the therapeutic potential of TSP-related pathways [10].

Given the expanding body of research and the emerging relevance of TSP-2 as both a mechanistic mediator and a promising biomarker in cardiovascular pathology, this review aims to provide a comprehensive summary of current knowledge regarding TSPs, with particular emphasis on TSP-2. We highlight their molecular mechanisms, clinical associations, diagnostic potential, and possible implications for risk stratification and therapeutic interventions in CVD.

**Table 1:** Overview of structural, functional, and clinical features of thrombospondins (TSP-1–TSP-5), summarizing gene localization, tissue distribution, biological processes, receptor binding profiles, potential clinical applications, and disease associations.

TSP Type	Gene Information	Localization	Biological Process	Receptors/ Binding Partners	Clinical Potential	Examples of Pathological Involvement	Literature
TSP-1 Thrombospondin 1	*THBS1* (15q15)	Platelets	Inhibition of angiogenesis; regulation of endothelial and smooth muscle cell migration; ECM interaction	CD36, CD47, integrins, LRP1	Anti-angiogenic therapeutic target; biomarker in CVDs	CAD, Atherosclerosis, hypertension, ischemic stroke	[11,12,13,14,15]
TSP-2 Thrombospondin 2	*THBS2* (6q27)	Fibroblasts, smooth muscle cells	Anti-angiogenic activity; modulation of ECM remodeling; inhibition of endothelial cell proliferation and migration	MMPs, collagen, integrins	Potential biomarker of fibrosis and aneurysm progression	HF, AAA, CAD, hypertension, stroke	[11,15,16,17,18,19,20,21]
TSP-3 Thrombospondin 3	*THBS3* (1q21)	Cardiac cells	Bone development; ECM organization	Collagens, ECM proteins	Potential relevance in skeletal disorders; possible cardiac remodeling marker	Osteosarcoma, cardiomyopathy, acute myocardial infarction	[22,23,24]
TSP-4 Thrombospondin 4	*THBS4* (5q13)	Endothelial and smooth muscle cells of large vessels	Regulation of angiogenesis; promotion of vascular inflammation and atherogenesis	Integrins, ECM components	Candidate marker of cardiac hypertrophy and vascular inflammation	CAD, cardiac hypertrophy, atherosclerosis,	[11,14,25]
TSP-5 Thrombospondin 5 (COMP, cartilage oligomeric matrix protein)	*COMP* (19p13.1)	Cartilage cells, connective tissue	Bone and cartilage ECM assembly; chondrocyte adhesion and differentiation	Collagen II, collagen IX, matrilin-3	Diagnostic biomarker in osteoarthritis; potential biomarker in metastatic cancers	Bladder cancer, osteoarthritis	[26,27,28]

## 2. Search Strategy and Selection Criteria

This narrative review was based on a structured literature search in PubMed, Scopus, and Web of Science to identify relevant studies on TSPs, with particular emphasis on TSP-2 in CVDs. The search included articles published between January 2015 and April 2026. A combination of Medical Subject Headings (MeSH) and free-text terms was applied, including “thrombospondin”, “TSP-2”, “extracellular matrix”, “cardiac remodeling”, “fibrosis”, “angiogenesis”, “heart failure”, and “coronary artery disease”.

Original research articles, systematic reviews, and meta-analyses addressing the structural, molecular, and clinical aspects of TSPs were considered eligible. Particular attention was given to studies exploring differences between TSP subgroups (trimeric TSP-1/2 vs. pentameric TSP-3/4/5) and their roles in cardiovascular pathophysiology, including endothelial function, ECM turnover, angiogenesis, and TGF-β-mediated fibrotic signaling.

Studies focusing on TSP-2 were prioritized, including experimental models (e.g., gene knockout studies) and clinical investigations evaluating its association with heart failure, ischemic risk, and adverse cardiovascular outcomes.

A total of 717 records were identified, 195 full-text articles were assessed for eligibility, and 120 studies were included in the final narrative synthesis.

Exclusion criteria comprised non-English publications, conference abstracts without full-text availability, case reports, and studies with limited methodological quality or relevance.

The final selection of articles was based on relevance, methodological rigor, and contribution to the understanding of TSP-2 as a mechanistic mediator and potential biomarker in CVD. As this review was not designed as a systematic review or meta-analysis, studies were selected based on their scientific relevance and contribution to the field rather than through a formal PRISMA-guided screening process.

## 3. Biological and Molecular Function of TSPs

**Figure 1:**
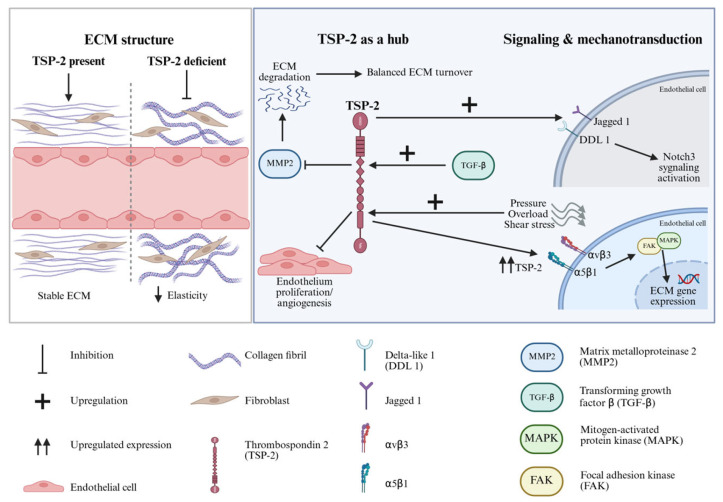
The role of TSP-2 in regulating extracellular matrix homeostasis, vascular signaling, and mechanotransduction. The left panel illustrates the effects of TSP-2 on ECM organization. Under physiological conditions, TSP-2 promotes the formation of organized collagen fibrils, whereas TSP-2 deficiency results in ECM disorganization, enlarged collagen fibrils, and reduced tissue elasticity. The central panel demonstrates the regulatory role of TSP-2 in matrix turnover and angiogenesis through the inhibition of MMP-2 activity and endothelial cell proliferation. TSP-2 expression is upregulated by TGF-β signaling. The right panel depicts the involvement of TSP-2 in mechanotransduction and intracellular signaling. Mechanical stimuli increase TSP-2 expression and modulate integrin- and FAK/MAPK-dependent pathways. In addition, TSP-2 enhances Notch3 activation in the presence of Jagged1 and DLL1, thereby contributing to vascular remodeling. Created in BioRender. Kazik, A. (2026) https://BioRender.com/fsm5un9 (accessed on 17 June 2026).

TSPs are ECM glycoproteins that play essential roles in matrix organization, cell signaling, and tissue remodeling. Among them, TSP-2 is abundantly expressed within the ECM and contributes to maintaining structural integrity and functional homeostasis, particularly in the cardiovascular system. The ECM not only provides mechanical support to cardiac and vascular tissues but also regulates key cellular processes, including proliferation, apoptosis, and repair following injury (Figure 1) [29,30].

TSP-2 functions as an important regulator of angiogenesis and vascular homeostasis. It inhibits endothelial cell proliferation and neovascularization, and its deficiency has been associated with increased vascular density in vivo. In contrast to TSP-1, TSP-2 lacks specific sequence motifs required for direct activation of latent TGF-β, highlighting functional divergence within the TSP family [7,31,32]. Nevertheless, TSP-2 plays a critical role in ECM organization. In TSP-2-deficient models, collagen fibrils exhibit increased size, irregular morphology, and broader distribution, while connective tissues such as those found in the skin display reduced elasticity [33,34,35,36]. These observations underscore the importance of TSP-2 in collagen fibrillogenesis and the maintenance of tissue mechanical properties [33,34].

At the molecular level, TSP-2 regulates ECM remodeling through interactions with matrix metalloproteinases (MMPs) and other ECM components. Through its N-terminal heparin-binding domain, TSP-2 modulates the activity and availability of proteases such as MMP-2, thereby influencing ECM turnover under both physiological and pathological conditions. Experimental studies have demonstrated that TSP-2 deficiency is associated with dysregulated MMP-2 and MMP-9 activity, contributing to impaired collagen fibrillogenic and structural vulnerability of the myocardium [37]. Furthermore, TSP-2 interacts with tissue transglutaminase-2 (tTG-2), an enzyme involved in collagen crosslinking and matrix stabilization, and modulates Src kinase-dependent signaling pathways that regulate cell–matrix adhesion and fibroblast activation. These interactions are particularly relevant in the context of cardiac protection: demonstrated that TSP-2 prevents cardiac injury in viral myocarditis by activating regulatory T cells, an effect mechanistically linked to TSP-2’s influence on ECM-immune cell crosstalk [38]. Collectively, these data highlight TSP-2 as a multifunctional matrix regulator whose disruption has severe structural and immunological consequences for the heart. This regulatory function is closely linked to TGF-β signaling, a central pathway controlling ECM synthesis and fibrosis. TGF-β upregulates TSP-2 expression, which in turn modulates MMP levels and ECM composition, contributing to the balance between matrix deposition and degradation. Disruption of this regulatory axis may lead to altered tissue stiffness and pathological remodeling [37,38].

Beyond its interactions with MMPs, TSP-2 may also contribute to ECM remodeling through functional interplay with members of the ADAMTS (a disintegrin and metalloproteinase with thrombospondin motifs) protease family. ADAMTS enzymes, particularly ADAMTS-1, ADAMTS-4, ADAMTS-5, and ADAMTS-7, are major regulators of proteoglycan turnover, versican cleavage, and vascular ECM remodeling and have been increasingly implicated in aortic aneurysm formation and atherosclerotic plaque instability [39]. Both TSP-2 and ADAMTS proteins contain thrombospondin type-1 repeat (TSR) domains that facilitate interactions with ECM components and proteoglycans. Although direct molecular interactions between TSP-2 and ADAMTS proteases remain incompletely characterized, their overlapping ECM-binding properties suggest potential functional convergence within the vascular wall [40]. Given the established role of TSP-2 in regulating MMP-2 activity and matrix organization, it is plausible that alterations in TSP-2 expression may indirectly influence broader proteolytic networks that include ADAMTS enzymes. This concept is particularly relevant in AAA, where dysregulated ADAMTS-1 expression and increased ADAMTS-7 levels have been reported and are associated with pathological ECM degradation and weakening of the aortic wall [41]. Further studies are required to determine whether TSP-2 directly modulates ADAMTS-dependent remodeling. However, the potential interaction between these pathways represents an emerging mechanism linking matrix homeostasis to vascular disease progression [7].

In addition to its role in ECM dynamics, TSP-2 participates in the regulation of intracellular signaling pathways. Both Notch-dependent and Notch-independent mechanisms have been described in this context. Experimental studies have demonstrated that TSP-2 enhances Notch3 signaling in the presence of its ligands, such as Jagged1 and Delta-like 1, without altering receptor expression levels [35,36]. Notably, this effect appears to be only specific to TSP-2, as TSP-1 does not exhibit similar activity, suggesting distinct, context-dependent interactions with vascular cells. Moreover, TSP-2 may influence additional signaling pathways independently of Notch, although these mechanisms remain incompletely understood [35,42].

TSP-mediated regulation of ECM remodeling is also closely associated with inflammatory processes and vascular pathology [43,44]. It has been shown that TSP-1 promotes the expression of pro-inflammatory cytokines and MMPs, facilitating ECM degradation and vascular cell migration [43,44]. In contrast, TSP-2 is upregulated in conditions such as aortic dissection and contributes to smooth muscle cell apoptosis through pro-inflammatory signaling pathways, including nuclear factor kappa-light-chain-enhancer of activated B cells (NF-κB) [43,45]. These findings indicate that dysregulation of TSP-dependent pathways may drive maladaptive ECM remodeling, vascular stiffening, and disease progression.

Recent studies have highlighted the role of TSPs as mediators of mechanotransduction in the cardiovascular system [1]. Under physiological conditions, TSP expression is relatively low; however, it is significantly upregulated in response to pathological mechanical stimuli, such as hypertension or pressure overload [29]. TSP-2, along with TSP-1 and TSP-4, appears to exert protective effects under these unfavorable conditions by supporting vascular integrity. Conversely, the absence of TSP-2 leads to structural defects, including reduced resistance to mechanical stress and disruption of vascular architecture [20,46].

Mechanistically, TSPs respond to mechanical cues such as stretch and shear stress by modulating integrin-mediated signaling pathways [47]. Activation of integrins, including αvβ3 and α5β1, triggers downstream signaling cascades involving focal adhesion kinase (FAK) and mitogen-activated protein kinases (MAPK), ultimately regulating the expression of ECM-related genes [47,48,49]. This process is particularly important in endothelial cells, fibroblasts, and vascular smooth muscle cells, where it enables adaptation to mechanical load [49]. However, dysregulation of these pathways may contribute to pathological conditions, including hypertension, vascular stiffness, and cardiac remodeling.

Collectively, TSPs emerge as multifunctional regulators of ECM organization, cell signaling, and tissue remodeling with a significant mechanobiological impact. Beyond their established roles in angiogenesis, inflammation, and growth factor signaling, TSPs act as dynamic mediators that match mechanical stimuli to cellular responses and matrix remodeling. This integrative function is especially relevant in the cardiovascular system, where continuous mechanical stress necessitates tightly controlled adaptive mechanisms to preserve tissue homeostasis.

## 4. Regulation of Thrombospondin Expression

The expression of TSPs, particularly TSP-1 and TSP-2, is tightly and dynamically regulated by a broad spectrum of biochemical and biomechanical stimuli. Key modulators include cytokines, hypoxic conditions, oxidative stress, and mechanical forces acting on the vascular wall. These factors play essential roles in maintaining cardiovascular homeostasis as well as in driving pathological processes such as vascular remodeling and disease progression. The complexity of regulatory mechanisms governing TSP expression reflects their significant biological functions and involvement in multiple signaling pathways [50].

### 4.1. Cytokine-Mediated Regulation

One of the most important regulators of TSP expression is TGF-β, a central mediator of ECM synthesis and fibrosis [51]. TGF-β upregulates TSP-1 and TSP-2 expression in multiple cell types, including fibroblasts, endothelial cells, and vascular smooth muscle cells. This creates a regulatory feedback loop, as TSP-1 is capable of activating latent TGF-β, thereby amplifying profibrotic signaling [52,53]. In contrast, TSP-2, which lacks the ability to directly activate TGF-β, modulates this pathway indirectly, contributing to ECM stabilization rather than amplification of fibrosis. Other cytokines, such as interleukin-1β (IL-1β) and tumor necrosis factor-α (TNF-α), have also been shown to regulate TSP expression, particularly under inflammatory conditions. These signals link TSPs to vascular inflammation, a key component of atherosclerosis and aneurysm formation [54,55].

### 4.2. Hypoxia and HIF-1α Signaling

Hypoxic conditions, commonly present in ischemic cardiovascular tissues, represent another important regulatory axis for TSP expression. Hypoxia-inducible factor-1α (HIF-1α) can modulate TSP-1 levels, although the direction of regulation appears to be context-dependent. In certain settings, hypoxia suppresses TSP-1 to promote angiogenesis [56,57], while in others it may contribute to vascular remodeling through altered ECM signaling [58]. Experimental studies in human cells support this duality: in human coronary artery smooth muscle cells (CASMCs), hypoxia-induced stabilization of HIF-1α leads to increased TSP-1 expression and secretion, promoting cell migration through ECM remodeling mechanisms. In contrast, in human microvascular endothelial cells (HMEC-1), hypoxia also upregulates TSP-1, but in this context TSP-1 exerts anti-angiogenic effects by inhibiting endothelial proliferation and inducing apoptosis despite concurrent activation of vascular endothelial growth factor (VEGF) -dependent pathways [56,57,58].

This dual role highlights the context-specific nature of TSP regulation and suggests that their levels may reflect local tissue oxygenation status, further supporting their potential as dynamic biomarkers.

### 4.3. Oxidative Stress

Oxidative stress, a hallmark of CVDs such as hypertension and HF, is another key modulator of TSP expression [7]. Reactive oxygen species (ROS) can induce TSP-1 expression via redox-sensitive transcription factors, including members of the FoxO family, which directly bind to the THBS1 promoter and regulate its transcription [59]. In endothelial cells, this mechanism contributes to stress-induced upregulation of TSP-1, thereby linking oxidative signaling to endothelial dysfunction and vascular inflammation [59].

Importantly, TSP-1 has been implicated in the inhibition of nitric oxide (NO) signaling through its interaction with CD47, contributing to impaired vasodilation [2,60,61]. Mechanistically, TSP-1/CD47 signaling suppresses endothelial nitric oxide synthase (eNOS) activity and sGC–cGMP signaling while promoting NADPH oxidase–dependent ROS generation, leading to reduced NO bioavailability and vascular dysfunction [62,63,64].

### 4.4. Mechanotransduction and Mechanical Forces

Mechanical stimuli, including shear stress, cyclic stretch, and pressure overload, are major regulators of TSP gene expression in cardiovascular tissues [1]. These forces activate mechanotransduction pathways involving integrins (e.g., αvβ3, α5β1), which in turn trigger intracellular signaling cascades such as focal adhesion kinase (FAK) and mitogen-activated protein kinase (MAPK) [65,66,67]. These pathways ultimately lead to changes in gene transcription, including upregulation of *THBS1* and *THBS2* genes, resulting in increased synthesis and deposition of TSP proteins in the ECM. In conditions such as hypertension or aortic stenosis, sustained mechanical stress significantly increases TSP expression, linking hemodynamic load to ECM remodeling and vascular stiffness. At the transcriptional level, mechanosensitive factors such as NF-κB, Activator Protein-1 (AP-1), and potentially Yes-associated protein/transcriptional coactivator with PDZ-binding motif (YAP/TAZ) are involved in mediating these responses, although their precise roles in TSP regulation remain an active area of investigation [47,68].

### 4.5. Micro-RNA-Mediated Regulation

Post-transcriptional regulation of TSP gene expression is mediated by microRNAs (miRNAs), which fine-tune protein levels in response to environmental cues [69]. Several miRNAs have been implicated in the regulation of *THBS1* and *THBS2* mRNA, including miR-18a, miR-194, and miR-221/222 [70,71,72], which are known to play roles in angiogenesis, inflammation, and vascular remodeling. Dysregulation of these miRNAs may lead to altered TSP protein expression in pathological conditions, further supporting the concept that TSPs are part of a broader regulatory network responsive to cardiovascular stress.

The tight regulation of TSP gene expression, particularly *THBS1* and *THBS2*, and subsequent protein synthesis by key pathological stimuli, including inflammation, hypoxia, oxidative stress, and mechanical load, underscores their relevance as biomarkers in CVDs. Because TSP protein levels integrate multiple signaling pathways, they may serve as sensitive indicators of ongoing vascular remodeling, ECM turnover, and tissue stress. Importantly, the dynamic and context-dependent regulation of *THBS1* and *THBS2* expression suggests that these molecules could provide not only diagnostic but also prognostic information, reflecting disease severity and progression, as well as responses to therapeutic interventions [29].

This multidimensional regulation highlights TSPs as promising biomarkers in CVDs and underscores their potential as therapeutic targets.

## 5. Thrombospondins in Cardiovascular Pathologies: From Mechanistic Insights to Biomarker Potential

### 5.1. The Concept of TSPs as Integrative Biomarkers

In modern medicine, biomarkers play a crucial role in both the diagnosis and the comparative assessment of disease progression, providing measurable values regarding the state of a specific organ [73]. For clinical application, an ideal biomarker must demonstrate reproducibility across different laboratories, high analytical sensitivity, and independence from inter-individual variables such as age or ethnic background [74]. However, substantial heterogeneity and dependence on multiple disease-related factors in CVDS complicate the establishment of standardized, independent markers [75].

TSPs, particularly TSP-1 and TSP-2, have emerged as important modulators of cardiovascular remodeling and excellent candidates for biomarker development because their expression is directly upregulated by tissue injury, inflammation, and mechanical stress, which are central features of CVDs. Rather than acting as markers of acute cardiomyocyte necrosis (such as troponins), TSPs function at multiple biological levels:

Protein-level biomarkers: Circulating levels in plasma reflect ongoing ECM turnover, fibrotic activity, and vascular stress [9,21,29].

Gene-level biomarkers: Local transcriptional activity (*THBS* mRNA expression and genetic polymorphisms) regulates protein availability and mirrors regional tissue responses [29,76,77].

Non-coding RNA regulators: miRNAs and long non-coding RNAs (lncRNAs) form complex epigenetic networks that fine-tune TSP expression during cardiovascular pathogenesis [70,78,79].

Through these interconnected levels, TSP-2 integrates signals from TGF-β–dependent fibrotic signaling, MMP regulation, and mechanotransduction pathways (including FAK/MAPK cascades) [9,29,46,80]. Consequently, its evaluation provides a biologically plausible reflection of active molecular remodeling.

### 5.2. Heart Failure

Heart failure (HF) is characterized by progressive myocardial remodeling, including fibrosis, cardiomyocyte hypertrophy, and impaired contractility, processes tightly linked to ECM turnover and profibrotic TGF-β signaling [81,82,83,84]. TSP-2 plays a crucial role in maintaining ECM integrity and modulating these fibrotic responses. The TGF-β/TSP-2/MMP axis is central to this process; TSP-2 is induced by TGF-β and subsequently regulates ECM composition by modulating MMP-2 activity, balancing matrix synthesis and degradation [37,46]. When this axis is dysregulated, excessive ECM deposition leads to increased myocardial stiffness and impaired cardiac function [37].

Clinically, elevated circulating levels of TSP-2 are consistently reported in HF patients and correlate with disease severity, risk of myocardial ischemia, and metabolic parameters such as insulin resistance and body weight [9,85]. Because TSP-2 reflects the intensity of structural myocardial remodeling rather than acute cardiomyocyte injury, it serves as a complementary marker to conventional natriuretic peptides [86]. Higher plasma TSP-2 concentrations correlate with an increased risk of hospitalization, disease progression, and mortality [87]. Crucially, the combined assessment of TSP-2 and N-terminal pro-B-type natriuretic peptide (NT-proBNP) significantly improves risk stratification and prognostic accuracy [83,87].

At the non-coding RNA level, epigenetic networks actively modulate this system. For instance, miR-221 exerts antifibrotic effects in the myocardium by directly targeting TSP-1, thereby inhibiting downstream TGF-β1 activation and profibrotic signaling during renal failure-induced cardiac fibrosis [70]. Additionally, several HF-associated miRNAs (such as miR-15-5p, miR-21-5p, miR-24-3p, miR-25-3p, miR-199b-5p, and miR-214-3p) show altered profiling in patients, mapping to the broad regulatory networks controlling ECM remodeling [78].

### 5.3. Coronary Artery Disease

Coronary artery disease (CAD) is driven by atherosclerosis, a chronic inflammatory process involving lipid accumulation, endothelial dysfunction, and vascular wall remodeling [88,89]. Plaque stability is strongly influenced by the balance between matrix synthesis and degradation [90,91]. Within this microenvironment, TSPs contribute to multiple aspects of atherogenesis, including smooth muscle cell (SMC) migration, inflammatory cell recruitment, and endothelial responses. TSP-2 modulates endothelial and SMC signaling through integrin-dependent pathways involving FAK and MAPK cascades, which are activated by disturbed flow and mechanical stress, promoting plaque progression [80,92]. Furthermore, while TSP-2 is less directly anti-angiogenic than TSP-1, it influences tissue perfusion in ischemic conditions by limiting local pro-angiogenic signaling [93].

From a biomarker perspective, increased circulating protein levels of TSP-2 are associated with a higher risk of adverse outcomes, including 3-year HF-related death, rehospitalization, and all-cause mortality [21]. Mirroring the trends seen in HF, combining TSP-2 measurements with NT-proBNP enhances the long-term prediction of adverse cardiovascular events and rehospitalization risk [83,94,95].

At the genetic level, polymorphisms in *THBS1* and *THBS4* variants have been linked to an increased risk of developing CAD [96]. In contrast, genetic studies on *THBS2* polymorphisms have yielded mixed and less consistent results regarding initial CAD susceptibility [76,96]. This suggests that circulating TSP-2 protein levels, rather than *THBS2* genetic variations alone, serve as a superior dynamic biomarker reflecting active disease progression and clinical risk [21]. This protein-level response is accompanied by distinct non-coding RNA signatures in CAD patients, such as the upregulation of miR-1-3p and miR-133a-3p, and the downregulation of miR-181b-5p [78].

### 5.4. Hypertension

Hypertension is characterized by chronic elevation of blood pressure, leading to vascular remodeling, increased arterial stiffness, and endothelial dysfunction [97]. Mechanical forces, such as shear stress and pressure overload, trigger adaptive and maladaptive changes in the vascular wall [98,99]. TSPs are inherently mechanosensitive proteins [1]. Chronic hemodynamic stress induces local TSP-2 expression via integrin-mediated FAK/MAPK activation, contributing to ECM reorganization [1,8,80]. While initially adaptive, this response ultimately facilitates maladaptive vascular stiffening and reduced vascular compliance [8].

Clinically, circulating TSP-2 protein levels are significantly higher in hypertensive individuals compared to normotensive controls. Moreover, plasma TSP-2 concentrations directly correlate with both systolic and diastolic blood pressure [9,20]. These findings indicate that circulating TSP-2 can serve as a novel biomarker reflecting vascular wall stress and subclinical remodeling, potentially aiding in the early detection and tracking of hypertensive vascular disease before overt organ damage occurs [9,20].

### 5.5. Abdominal Aortic Aneurysm and Aortic Dissection

Abdominal aortic aneurysm (AAA) and aortic dissection are characterized by progressive weakening of the aortic wall resulting from excessive ECM degradation, chronic inflammation, and vascular smooth muscle cell (VSMC) apoptosis [76,100]. Dysregulation of MMPs and loss of structural elastic fibers are hallmark features of these conditions [41]. TSP-1 and TSP-2 are vital regulators of ECM turnover and inflammatory cell signaling within the aortic wall [77,101]. TSP-2 deficiency in experimental models impairs collagen fibrillogenesis, rendering the aorta highly susceptible to structural failure [34]. Mechanistically, TSP-2 influences the VSMC phenotype and suppresses excessive matrix degradation by modulating MMP-2/9 activity [77,102] (Table 2).

At the gene expression level, spatial analyses of AAA tissues reveal a highly coordinated transcriptional network along the aneurysm structure. Both *THBS1* and *THBS2* mRNA levels are highly enriched in the aneurysm border and the aneurysm sac, where they are primarily expressed by infiltrating macrophages, linking local transcription to active inflammatory remodeling [77,102]. Concurrently, *THBS3* expression decreases from the border to the sac but remains positively correlated with *THBS1* and *THBS2* transcription [77,102].

Clinically, increased expression of TSP-2 is observed in diseased aortic tissues and cases of aortic dissection [43]. Higher circulating TSP-2 protein levels correlate with larger aneurysm size, faster growth rates, and disrupted vascular wall integrity [77]. Consequently, circulating TSP-2 represents a promising integrative biomarker for identifying patients at high risk for rapid aneurysm expansion or catastrophic structural complications.

This localized vascular failure is heavily regulated by non-coding RNAs; multiple miRNAs implicated in AAA pathogenesis (such as miR-24, miR-29, and miR-145) modulate matrix phenotypes [79]. Furthermore, a competing endogenous RNA (ceRNA) network has been described where the long non-coding RNA LINC01197 functions as a molecular sponge for miR-150 [103]. Because miR-150 directly suppresses TSP-2 expression, its sequestration by LINC01197 drives the upregulation of TSP-2, establishing a clear link between non-coding RNA activity, gene regulation, and protein availability during vascular remodeling [103].

**Table 2:** Summary of thrombospondin-related biomarkers by cardiovascular pathology.

Cardiovascular Pathology	Protein-Level Findings (Circulating/Tissue)	Gene-Level and mRNA Findings	Non-Coding RNA Regulators	Clinical Utility and Significance	Key Clinical Statistics (Validated Cohort Data)	Literature
Heart Failure (HF)	Elevated plasma TSP-2 levels. Reflects myocardial fibrosis intensity over acute injury.	*THBS2* transcriptional activity regulates structural tissue availability.	Altered profiles of miR-15-5p, miR-21-5p, miR-24-3p, miR-25-3p, miR-199b-5p, miR-214-3p, and miR-148a-3p. miR-221 directly targets TSP-1 to reduce fibrosis.	Predicts risk of hospitalization, disease progression, and mortality. Combines with NT-proBNP to improve prognostic accuracy and risk stratification.	Cut-off: 14.65 ng/mL; ROC-AUC: 0.823; sensitivity 89.6%; specificity 63.3%. OR all-cause mortality: 1.12 (95% CI 1.07–1.17); OR HF-related death: 1.08 (95% CI 1.02–1.11); OR HF rehospitalization: 1.10 (95% CI 1.02–1.15).	[9,21,29,43,70,78,83,85,86,87]
Coronary Artery Disease (CAD)	Elevated plasma TSP-2. Local TSP-2 levels associated with plaque remodeling dynamics.	*THBS1* and *THBS4* polymorphisms increase susceptibility. *THBS2* polymorphisms show inconsistent association with initial risk.	Increased expression of miR-1-3p and miR-133a-3p. Decreased ex-pression of miR-181b-5p.	Predicts 3-year HF-related mortality, all-cause mortality, and rehospitalization. Synergizes with NT-proBNP to enhance long-term predictive performance.	Among CAD patients with CHF: OR all-cause mortality: 1.27 (95% CI 1.08–1.59); OR CHF-related death: 1.16 (95% CI 1.02–1.50); OR rehospitalization: 1.12 (95% CI 1.07–1.25). Cut-off: 14.65 ng/mL; ROC-AUC: 0.823.	[21,43,76,78,83,94,95,96]
Hypertension	Significantly increased circulating TSP-2 levels.	Mechanosensitive upregulation of *THBS2* transcription due to hemodynamic overload.	Modulated by downstream mechanosensitive RNA networks.	Plasma concentration directly correlates with systolic and diastolic blood pressure. Serves as a novel marker for early detection and tracking of subclinical vascular stress.	No validated prospective HR/OR, ROC-AUC or clinical cut-off values currently available in human cohorts; evidence remains mainly associative.	[1,9,20]
Abdominal Aortic Aneu-rysm (AAA) and Aortic Dissec-tion	Elevated TSP-2 in diseased aortic tissues. Correlates with elastin degradation and MMP-2/9 activity.	High *THBS1* and *THBS2* mRNA expression in aneurysm border and sac (mainly via macrophages). *THBS3* transcription correlates positively with *THBS1*/*THBS2*.	Implicated miRNAs: miR-145, miR-24, miR-33, miR-125, let-7, miR-15, miR-191, miR-29, and miR-133. lncRNA LINC01197 sponges miR-150 to upreglate TSP-2.	Correlates with larger aneurysm size, accelerated growth rate, and impaired vascular wall integrity. Potential indicator for high risk of structural progression or complication.	Human studies demonstrate significant elevation and correlation with TNF-α and IL-6; however, validated prognostic HR/OR values and ROC-derived cut-offs are currently unavailable	[38,77,87,102,103]

## 6. Potential Therapeutic Strategies Targeting TSP-2-Related Pathways

Therapeutic strategies related to TSP-2 can be conceptualized as interventions aimed at modulating ECM remodeling, vascular integrity, and fibrotic signaling networks through direct or indirect mechanisms. Current approaches remain at the experimental or preclinical stage and are primarily focused on regulating *THBS2* expression or interfering with downstream pathways involved in matrix turnover and tissue remodeling [104].

A critical consideration in the development of TSP-2–related therapies is the predominantly cardioprotective role of this matricellular protein. Experimental evidence suggests that TSP-2 is essential for maintaining myocardial structural integrity and regulating adaptive tissue remodeling. Swinnen et al. (2009) [37] demonstrated that TSP-2 deficiency results in age-related dilated cardiomyopathy, highlighting its importance in preserving myocardial ECM homeostasis. Similarly, Papageorgiou et al. (2012) [38] showed that TSP-2 protects against cardiac injury in viral myocarditis through activation of regulatory T cells. Moreover, genetic deletion of TSP-2 has been associated with increased susceptibility to cardiac rupture following pressure overload. Collectively, these findings indicate that elevated circulating TSP-2 levels observed in HF and related cardiovascular disorders may reflect a compensatory adaptive response rather than a direct pathogenic mechanism. Consequently, indiscriminate inhibition of TSP-2 is not supported by current experimental evidence and could potentially have deleterious effects on cardiac structure and function [93,104,105].

In light of these observations, therapeutic strategies are more appropriately directed toward modulation of the broader ECM remodeling network in which TSP-2 participates rather than toward complete suppression of TSP-2 activity. One indirect approach involves targeting upstream regulatory pathways that govern *THBS2* expression. In particular, interventions affecting transcriptional regulators and profibrotic signaling cascades, including transforming growth factor-β (TGF-β)-dependent pathways, may influence TSP-2 expression as part of a broader normalization of ECM turnover and fibrotic responses [106,107].

Another therapeutic avenue involves modulation of downstream effectors associated with TSP-2–mediated remodeling processes. Among these, MMPs, which play a central role in ECM degradation and renewal, represent attractive targets for restoring balanced matrix turnover. Likewise, interventions directed at integrin-mediated signaling and mechanotransduction pathways may indirectly influence ECM organization and tissue remodeling in ways that overlap functionally with TSP-2–associated mechanisms [108,109,110].

An additional concept focuses on context-dependent modulation rather than complete inhibition of TSP-2–related pathways. Given the complexity and interconnectivity of ECM regulatory networks, partial, temporally controlled, or tissue-specific modulation may be preferable to avoid disruption of physiological remodeling processes. This consideration is particularly relevant for emerging gene-silencing technologies and antibody-based approaches, where advances in targeted delivery systems may improve therapeutic selectivity while minimizing off-target effects.

Recent therapeutic perspectives increasingly emphasize systems-level ECM normalization strategies, in which TSP-2 is viewed as one component of a broader regulatory network. Combination approaches integrating modulation of *THBS2* expression with therapies targeting collagen cross-linking, matrix-degrading enzymes, or key profibrotic signaling pathways are being explored as potential means of restoring ECM homeostasis while preserving essential physiological remodeling functions.

Overall, therapeutic development targeting TSP-2–related pathways is evolving toward multi-level intervention strategies that integrate gene regulation, selective protein modulation, and broader control of ECM regulatory networks. Future approaches are likely to place increasing emphasis on biological specificity, temporal regulation, and tissue-targeted delivery in order to maximize therapeutic benefit while minimizing interference with the protective functions of TSP-2.

## 7. Limitations and Future Directions

### 7.1. Limitations of Current Evidence

The current evidence base linking TSP-2 to cardiovascular outcomes is primarily limited by study scale and design. Most available clinical investigations have been conducted in relatively small, often single-center cohorts, which restricts the generalizability of reported associations, effect sizes, and proposed diagnostic thresholds [9].

A further limitation is the absence of standardized protocols for TSP-2 quantification. Variability in biological material (serum versus plasma), assay platforms, and pre-analytical handling procedures complicates cross-study comparisons and prevents the establishment of universally applicable reference ranges [21].

In addition, circulating TSP-2 lacks disease specificity. Elevated levels have been observed in multiple pathological conditions associated with ECM remodeling, including pulmonary fibrosis, liver disease, and rheumatoid arthritis. As a result, TSP-2 should be interpreted as a marker of systemic tissue remodeling rather than a cardiovascular-specific biomarker, and its diagnostic utility depends on integration with clinical context and complementary biomarkers [111,112,113].

Evidence regarding ncRNA regulators of *THBS2* is also limited. Although miRNAs and lncRNAs have been proposed as upstream regulators of TSP-2 expression, most findings originate from non-cardiovascular models, and their translational relevance to cardiac pathology remains insufficiently validated. In addition, reliable quantification of circulating ncRNAs is still challenged by issues related to stability, low abundance, and lack of standardized normalization strategies [21].

Finally, therapeutic targeting of TSP-2-related pathways remains at a preclinical stage. No cardiovascular-specific pharmacological agents directly targeting *THBS2* have progressed to clinical evaluation. This reflects both limited mechanistic validation in human cardiovascular disease and uncertainty regarding systemic effects of modulating a matricellular protein involved in ECM homeostasis, angiogenesis, and tissue repair [21].

A major translational barrier is therefore the risk of off-target effects associated with systemic modulation of TSP-2. Global inhibition may disrupt physiological remodeling processes across multiple organs, raising important safety concerns. Although experimental strategies such as nanoparticle-based delivery systems and adeno-associated virus (AAV)-mediated gene modulation have been proposed to enable tissue-specific targeting, these approaches remain experimental in cardiovascular applications and are limited by challenges in biodistribution control, immunogenicity, and long-term expression stability [21].

Collectively, these limitations highlight a substantial gap between mechanistic insights into TSP-2 biology and its clinical translation.

### 7.2. Methodological and Translational Challenges

Several methodological challenges must be addressed before TSP-2 can be reliably implemented in clinical practice. A key priority is analytical standardization, including harmonization of immunoassay platforms, sample processing protocols, and reporting units. Without such standardization, inter-laboratory reproducibility and regulatory validation will remain limited.

Assay sensitivity represents another important limitation. The development of high-sensitivity methods capable of detecting low circulating concentrations of TSP-2, particularly in early disease stages, is essential for improving its clinical utility.

The integration of ncRNA-based biomarkers into clinically applicable workflows also remains technically challenging. Robust and reproducible detection platforms, as well as standardized normalization strategies, are required before ncRNA-mediated regulatory networks can be incorporated into cardiovascular risk assessment.

From a translational perspective, the lack of validated cardiovascular-specific delivery systems remains a major obstacle. Although nanoparticle-based and viral vector-based strategies are under active investigation, their clinical applicability is currently limited by insufficient targeting precision, safety concerns, and variability in delivery efficiency.

### 7.3. Future Perspectives

Several research directions are critical for translating current findings into clinical practice. The most immediate priority is large-scale prospective validation. Multicenter cohort studies enrolling patients across the full spectrum of CDV—stratified by HF phenotype (HFrEF vs. HFpEF), CAD severity, hypertension stage, and aneurysm size—are needed to establish robust reference intervals and outcome-linked thresholds for circulating TSP-2 [7].

A promising near-term direction is the integration of TSP-2 into multi-marker diagnostic and prognostic panels [114]. Its additive value to established biomarkers such as NT-proBNP supports its potential role within broader models incorporating markers of inflammation, fibrosis, and ECM turnover [115]. These approaches may be further enhanced by machine learning-based risk stratification strategies.

Mechanistic studies using cardiovascular-specific experimental models remain essential to clarify the biological role of TSP-2. Key unresolved questions include its involvement in endothelial-to-mesenchymal transition, its interaction with ECM remodeling pathways, and its modulation by therapies such as SGLT2 inhibitors and sacubitril/valsartan. Preliminary findings suggest potential biological interactions, although these require systematic validation in disease-relevant models [116,117].

Further research into ncRNA-mediated regulation of THBS2 should focus on cardiovascular tissue contexts. Regulatory networks described in other disease settings, such as the LINC01197–miR-150–THBS2 axis, require validation in cardiac and vascular systems. The identification of stable, circulating miRNAs directly targeting TSP-2 would significantly enhance its diagnostic and prognostic applicability [103,118,119].

Finally, therapeutic targeting of TSP-2-related pathways warrants systematic preclinical evaluation in CVD models. Future studies should assess tissue-selective and context-dependent modulation strategies, including nanoparticle-mediated siRNA delivery and antibody-based approaches, in models of pressure overload, ischemic injury, and aneurysm progression. Particular attention should be given to preserving the physiological roles of TSP-2 in extracellular matrix homeostasis while minimizing systemic exposure.

Collectively, these directions reflect the transition of TSP-2 research from associative clinical observations toward mechanistic and translational maturity. Rather than functioning as a standalone biomarker, TSP-2 is likely to achieve clinical relevance within integrated multi-marker frameworks that combine protein biomarkers, gene regulatory networks, and advanced computational risk models, pending robust prospective validation.

## 8. Conclusions

TSP-2 is a biologically grounded marker of cardiovascular ECM remodeling that reflects fibrotic and structural disease activity rather than acute myocardial injury. Current evidence supports its prognostic value in HF, where it adds incremental risk stratification to NT-proBNP by capturing fibrosis-related pathways not reflected by natriuretic peptides. Emerging data also suggest potential relevance in CAD, hypertension, and aortic aneurysm, although these findings remain preliminary. At the molecular level, regulation of THBS2 by ncRNAs represents an additional gene control mechanism that may play a role in the pathogenesis of CVDs. These mechanisms are not yet available, and there is growing evidence of their potential diagnostic and prognostic value in the future. Overall, TSP-2 shows promise as part of multi-marker cardiovascular risk models, but clinical translation will require large-scale validation and methodological standardization.

## Data Availability

No new data were created or analyzed in this study. Data sharing is not applicable to this article.

## Abbreviations

The following abbreviations are used in this manuscript:

AAA	Abdominal aortic aneurysm
AP-1	Activator Protein-1
CAD	Coronary artery disease
CASMC	Coronary artery smooth muscle cell
CVD	Cardiovascular disease
DLL-1	Delta-like ligand 1
ECM	Extracellular matrix
eNOS	Endothelial nitric oxide synthase
FAK	Focal adhesion kinase
HF	Heart failure
HFpEF	Heart failure with preserved ejection fraction
HFrEF	Heart failure with reduce ejection fraction
HIF-1α	Hypoxia-inducible factor-1α
HMEC-1	Human microvascular endothelial cells
IL-1β	Interleukin-1β
LncRNA	Long non-coding RNA
LRP1	Low-density lipoprotein receptor-related protein 1
MAPK	Mitogen-activated protein kinase
MeSH	Medical Subject Headings
MMP	Matrix metalloproteinase
NADPH	Nicotinamide adenine dinucleotide phosphate
ncRNA	Non-coding RNA
NF-κB	Nuclear factor kappa-light-chain-enhancer of activated B cells
NO	Nitric oxide
NT-proBNP	N-terminal pro-B-type natriuretic peptide
ROS	Reactive oxygen species
SDS-PAGE	Sodium dodecyl sulfate–polyacrylamide gel electrophoresis
TAZ	Transcriptional coactivator with PDZ-binding motif
TGF-β	Transforming growth factor beta
THBS	Thrombospondin gene
TNF-α	Tmor necrosis factor-α
TSP	Thrombospondin
VEGF	Vascular endothelial growth factor
YAP	Yes-associated protein
YAP/TAZ	Yes-associated protein/transcriptional coactivator with PDZ-binding motif
